# Subgingival Microbial Profiles at Peri-Implant and Adjacent Tooth Sites: A Cross-Sectional Quantitative PCR Study

**DOI:** 10.3390/dj14070394

**Published:** 2026-06-29

**Authors:** Ioana Suciu, Simona Ruță, George Suciu

**Affiliations:** 1 Romanian Academy, School of Advanced Studies of the Romanian Academy (SCOSAAR), Calea 13 Septembrie 13, 050711 Bucharest, Romania; simona.ruta@umfcd.ro; 2 Clinical Department, Carol Davila University of Medicine, 020021 Bucharest, Romania; 3 Stefan S. Nicolau’s Institute of Virology, 030304 Bucharest, Romania; 4 BEIA Consult International S.R.L., Research and Development Department, 18–22 Peroni Street, 041386 Bucharest, Romania; george@beia.eu

**Keywords:** peri-implantitis, peri-implant adjacent teeth, microbiota, bacterial species

## Abstract

**Background** Peri-implant diseases are primarily driven by microbial biofilm accumulation on the implant surface, leading to inflammatory destruction of the soft and hard tissues supporting dental implants, with progressive marginal bone loss and potential implant failure. **Objectives:** To analyze the subgingival microbial profiles of peri-implant sites and adjacent teeth in peri-implantitis and to assess the modifying effects of smoking and periodontal status. **Methods:** In this cross-sectional study, 43 adults (mean age: 54.5 years) contributed 100 dental implants and 50 adjacent natural teeth. Implants were classified as healthy implants (HI; n = 74) or peri-implantitis sites (PI; n = 26), and sampled teeth (TT) were categorized as periodontally diseased (n = 19) or periodontally healthy (n = 31). Subgingival biofilm was collected with sterile paper points and analyzed by quantitative polymerase chain reaction using the Paro x panel (ADD Laboral), which reported qualitative and quantitative data for 22 bacterial taxa. Additional subgroup analyses were performed according to smoking and periodontal status. **Results**: Peri-implantitis sites showed a significantly greater bacterial load than adjacent teeth for 10 taxa after false discovery rate correction, including *Campylobacter rectus* (β = 1.292, 95% CI 0.773–1.811; adjusted *p* = 2.38 × 10^−5^) and *Porphyromonas gingivalis* (β = 1.374, 95% CI 0.730–2.019; adjusted *p* = 3.22 × 10^−4^). Additional significant increases at peri-implantitis sites were observed for the *Streptococcus constellatus* group, *Fusobacterium nucleatum*, *Eubacterium nodatum*, *Filifactor alocis*, *Parvimonas micra*, *Tannerella forsythia*, *Actinomyces odontolyticus*, and *Treponema denticola*. In contrast, no taxon differed significantly in bacterial load between HI and TT after multiple-testing correction, and no taxon remained significant in prevalence analyses for either PI versus TT or HI versus TT. Clinically, significantly higher proportions of bleeding on probing and suppuration were present in PI sites vs. HI sites (96.15% of PI sites compared with 28.38%, 2.7%, 18.00%, and 8.00% of TT sites, respectively). Subgroup analyses by smoking and periodontal status suggested additional variation in microbial overlap, but these findings should be interpreted with caution given the limited subgroup sizes, particularly among smokers. **Conclusions:** Peri-implant and adjacent tooth sites shared substantial microbiological overlap; however, peri-implantitis sites showed higher detection frequencies and bacterial loads for selected taxa, whereas comparisons between healthy implant and tooth sites were less consistent.

## 1. Introduction

Peri-implant diseases represent a distinct category of contemporary oral infections that have emerged following the widespread clinical adoption of osseointegrated dental implants. These conditions, primarily driven by microbial biofilm accumulation on the implant surface, are characterized by inflammatory destruction of the soft and hard tissues supporting dental implants, leading to progressive marginal bone loss and potential implant failure. Peri-implant mucositis and peri-implantitis are considered biological complications that closely parallel gingivitis and periodontitis, respectively, within the context of natural dentition [1,2].

Peri-implantitis has a multifactorial etiology, arising from complex interactions among microbial biofilm, the host immune response, and environmental and systemic risk factors. Microbial pathogens may contribute to tissue destruction either by expressing local virulence factors or by perpetuating chronic subclinical inflammation. Identifying key etiological factors—including specific bacterial and viral agents, host genetic susceptibility, systemic comorbidities, and lifestyle—is critical to advancing targeted prevention, early diagnosis, and effective management strategies [3].

Site-specific variations in biofilm composition, modulated by implant location and dentition status, further support the hypothesis that local anatomical and ecological factors significantly influence patterns of bacterial colonization.

The bacterial composition associated with peri-implantitis has been extensively investigated over the past two decades. Initial research focused primarily on identifying conventional periodontal pathogens. However, the advent of next-generation sequencing technologies has revealed a far more complex and diverse microbial ecosystem than previously recognized [4,5].

Several authors have directly compared the subgingival microbiota of peri-implantitis with that of periodontitis. In a systematic review of diseased periodontal and peri-implant sulci, Rajasekar and Varghese found a broad overlap in the species detected at the two sites, together with differences in their relative proportions, and concluded that a bacterial profile clearly distinguishing the two conditions has not yet been established [6]. Earlier culture- and PCR-based reviews and meta-analyses reached a similar conclusion: no microbial signature unique to peri-implantitis could be identified, and classical periodontal pathogens such as *Porphyromonas gingivalis*, *Tannerella forsythia*, and *Aggregatibacter actinomycetemcomitans* were recovered from periodontitis, peri-implantitis, and, at lower levels, healthy implant sites [1,7]. At the clinical level, a systematic review, meta-analysis, and trial-sequential analysis of prospective cohort studies established a history of periodontitis as a significant risk factor for implant failure and peri-implantitis [8], reinforcing the biological link between the two diseases. Taken together, this body of work indicates that peri-implantitis and periodontitis share a largely common pathogenic flora while leaving open whether the diseased implant is colonized by a genuinely distinct community or by an altered quantitative balance of the same organisms—the question that the present within-patient design was intended to address.

The relationship between the microbiota at diseased implant sites and that of adjacent natural dentition is clinically significant, as it may provide important insights into the mechanisms underlying disease transmission and progression. Periodontally compromised teeth may function as microbial reservoirs, contributing to the initiation and progression of peri-implant diseases in partially dentate patients. Nevertheless, it remains unclear whether peri-implantitis represents a direct extension of periodontal infection or a distinct dysbiotic condition influenced by implant-specific ecological factors.

This study aimed to compare the subgingival microbial profiles of peri-implant sites and adjacent teeth in peri-implantitis and to explore whether smoking and periodontal status affect the microbiota of these neighboring sites.

## 2. Materials and Methods

### 2.1. Study Design and Patient Recruitment

This cross-sectional study was conducted in accordance with the ethical principles of the Declaration of Helsinki (World Medical Association), with all procedures carried out in a private clinical practice and approved by the Ethics Committee of BEIA (protocol code 7/2020, date of approval: 7 January 2020. The study was designed and is reported in accordance with the STROBE recommendations for cross-sectional studies. No formal a priori sample size calculation was performed; a convenience sample was used, comprising all eligible and willing patients available during the recruitment period.

Before enrolment, all participants received comprehensive information regarding the study objectives and potential benefits and provided written informed consent. Completely anonymized personal data were processed in accordance with the General Data Protection Regulation (GDPR). Each participant filled in a medical history and health status questionnaire, an informed consent for the collection, storage, and use of biological samples, and participation in the clinical study. In addition, consent for the processing of personal data, in accordance with the General Data Protection Regulation (GDPR), was obtained

Inclusion criteria comprised individuals aged 18 years or older, in general systemic health, presenting with at least one functional dental implant. Exclusion criteria included systemic antibiotic use within the past 6 months, chronic systemic diseases, pregnancy or lactation, implant mobility, functional implant loading of less than 1 year, and recent periodontal or peri-implant surgical interventions within the previous 12 months.

### 2.2. Clinical Examination

Comprehensive clinical examinations were conducted to assess peri-implant and periodontal status. The following parameters were recorded for each participant: age, gender, dentition status (fully edentulous or dentate), smoking habits, implant location (maxillary/mandibular; anterior/posterior), duration of functional loading, probing pocket depth (PPD), bleeding on probing (BoP), suppuration (SuP), and oral hygiene status. Clinical measurements were taken at one site per implant or tooth (buccal, mesial, distal, or lingual), selected as the site with the greatest probing depth. To keep the two site types comparable within the same analysis, we relied on the clinical inflammatory and pocket parameters applicable to both.

Intraoral periapical radiographs were obtained at a certified radiology center for the evaluation of marginal bone levels. Peri-implant health and disease diagnoses were established in accordance with the 2017 World Workshop on the Classification of Periodontal and Peri-Implant Diseases and Conditions [9] Peri-implantitis (PI) was defined as the presence of clinical signs of inflammation (BoP and/or SuP), a PPD ≥ 6 mm, and radiographic bone loss ≥ 3 mm from the implant shoulder.

For data analysis, sites with PPD < 5 mm were considered shallow, and those with PPD ≥ 5 mm were considered deep. When both healthy and diseased implants were present in a single patient, classification was based on the most severe diagnosis, and the subject was assigned to the peri-implantitis group. This patient-level assignment was used only for descriptive and baseline comparisons. The microbiological analyses were performed at the site level so that each implant retained its own diagnosis (healthy implant or peri-implantitis) and contributed individually to the models; healthy and diseased implant sites belonging to the same patient were therefore both included and directly comparable, with the within-patient correlation handled by the random-effects structure described in Section 2.5. Because peri-implantitis is more difficult to identify, in patients with both peri-implantitis and healthy implants, priority was given to sampling the implants with peri-implantitis. Sampled natural teeth (TT) were classified as periodontally diseased when the sampled site showed clinical evidence of periodontal inflammation and breakdown, defined as increased probing depth with bleeding on probing; radiographic signs of periodontal support loss were also considered when present. Sampled teeth that did not meet these criteria were classified as periodontally healthy. At the patient level, participants were categorized as having periodontal disease when at least one sampled natural tooth met the criteria for periodontal disease; otherwise, they were classified as periodontally healthy. This represents a pragmatic, sample-based operational definition adopted for the purposes of the site-level microbiological comparison. It does not correspond to the full 2017 World Workshop (AAP/EFP) periodontitis case definition, which requires interproximal clinical attachment loss at two or more non-adjacent teeth. Because the study used a single-site sampling protocol rather than full-mouth periodontal charting, clinical attachment level was not recorded; patient-level periodontal status should therefore be interpreted as an approximation, and this limitation is acknowledged in the Discussion.

Because the study was designed as a site-level microbiological comparison, one sampling site per implant or tooth was selected. The site with the greatest probing depth was chosen to represent the location with the highest presumed inflammatory and microbial burden, thereby maximizing the likelihood of detecting clinically relevant dysbiotic changes. This standardized approach improved comparability across sampled units but does not represent a full-mouth periodontal or peri-implant assessment.

### 2.3. Collection of Peri-Implant and Tooth Samples

For each participant, a customized collection kit was used. The kit included four color-coded 1.5 mL screw-cap microtubes (Sarstedt, Nümbrecht, Germany) in blue, yellow, orange, and black, as well as sterile paper points (#50, Spident, Incheon, Republic of Korea). Each microtube was assigned to collect samples from either a dental implant or a natural tooth. The clinical condition of each implant was assessed and categorized as either a healthy implant (HI) or peri-implantitis (PI). Patients were instructed to abstain from eating, drinking, and performing oral hygiene procedures for at least 30 min before sample collection. Before microbiological sampling, the selected sites were carefully isolated with cotton rolls to minimize the risk of salivary contamination. Rubber-dam isolation was not employed because placing a clamp at the gingival or peri-implant margin would risk mechanically disturbing and contaminating the precise subgingival site to be sampled. Cotton-roll isolation, combined with air drying and supragingival plaque removal, is an established and widely used approach for subgingival paper-point sampling. Supragingival plaque was gently removed using a curette or a stream of compressed air. Subgingival plaque samples were then collected using sterile paper points. Throughout the procedure, particular care was taken to avoid inducing sulcular bleeding and to ensure the integrity of the microbial samples. Paper points were inserted gently to minimize iatrogenic trauma. At sites with bleeding on probing, some sulcular bleeding was unavoidable; in these cases, care was taken to minimize gross blood contamination and preserve the integrity of the microbial samples. Clinical parameters and actual sampling were recorded in separate sessions to minimize bleeding.

Peri-implant crevicular fluid samples were collected from one predetermined site per implant or tooth (buccal, lingual/oral, mesial, or distal) using five sterile paper points. Each paper point was inserted and maintained in place for 15 s to ensure adequate sample absorption. Subsequently, all five paper points were pooled and placed into the corresponding color-coded microtube designated for that site.

Patients were sampled according to a standardized protocol, with a maximum of 4 sites per individual. At least one site corresponded to an adjacent natural tooth. In cases diagnosed with peri-implantitis, the tooth adjacent to the affected implant was specifically selected for comparative sampling.

If a participant presented with multiple implants exhibiting the same peri-implant disease status, submucosal biofilm samples were collected from two to a maximum of three distinct implant sites per individual. Additionally, sampling from a neighboring natural tooth was mandatory in each case to allow for intra-subject comparison. Following collection, the biological samples were shipped via postal service to the designated laboratory, where DNA was extracted from both peri-implant and periodontal crevicular fluid samples.

### 2.4. qPCR Analysis

Bacterial identification and quantification of the 22 target taxa were not performed in-house. Subgingival samples were analyzed for microbial content by an external commercial laboratory (ADD Laboral B.V./Advanced Dental Diagnostics, Malden, The Netherlands) using the Perio 22 species-specific quantitative real-time PCR panel. Following collection, samples were shipped to the laboratory under routine conditions. DNA was extracted with InstaGene Matrix (Bio-Rad Laboratories, Hercules, CA, USA), a Chelex-100–based resin, according to the manufacturer’s instructions. For crevicular fluid samples, 150 µL of InstaGene Matrix was added directly to the Eppendorf tube containing the pooled paper points. The extracted template was then analyzed with the Perio 22 panel, a species-specific real-time PCR assay that detects and quantifies 22 periodontitis-associated taxa, with results reported by the laboratory as both qualitative (presence/absence) and quantitative (colony-forming-unit equivalents per sample). Detection, amplification, and quantification were performed by the manufacturer’s laboratory using its validated proprietary primer/probe assays; the specific primer and probe sequences are not publicly disclosed by the manufacturer. The performance characteristics of this commercial panel have been independently evaluated against other periodontal qPCR kits [10]. The assay’s primer/probe sequences and internal cycling parameters are proprietary to the manufacturer and are not publicly released, as is the case for other commercial periodontal qPCR systems on the market [10]. Because microbial analysis relied on a proprietary commercial qPCR panel, assay-level parameters such as primer/probe sequences and limits of detection were not available to the authors.

The panel uses quantitative PCR (qPCR) to detect bacterial DNA in clinical samples. It targets 22 specific periodontal pathogens, namely: *Aggregatibacter actinomycetemcomitans* (Aa), *Porphyromonas gingivalis* (Pg), *Tannerella forsythia* (Tf), *Treponema denticola* (Td), *Fusobacterium nucleatum* (Fn), *Prevotella intermedia* (Pi), *Filifactor alocis* (Fa), *Parvimonas micra* (Pm), *Prevotella nigrescens* (Pn), *Eubacterium nodatum* (En), *Campylobacter rectus* (Cr), *Campylobacter gracilis* (Cg), *Eikenella corrodens* (Ec), *Campylobacter concisus* (Cc), *Capnocytophaga species* (Cs), *Actinomyces odontolyticus* (Ao), *Veillonella parvula* (Vp), *Actinomyces viscosus* (Av), *Streptococcus mitis group* (Smg), *Streptococcus constellatus* (Scg), *Streptococcus gordonii* (Sg), and *Enterococcus faecalis* (Ef). The assay provided both qualitative (presence/absence) and quantitative results with bacterial load expressed as colony-forming units (CFU), determined digitally based on qPCR amplification data.

### 2.5. Statistical Analysis

Statistical analyses were performed using IBM SPSS Statistics version 31 (IBM Corp., Armonk, NY, USA). Continuous variables were summarized as mean ± standard deviation or median and interquartile range, as appropriate, and categorical variables as counts and percentages. Because multiple sites could be sampled from the same participant, all site-level inferential analyses accounted for within-patient clustering. Continuous outcomes were assessed using the Shapiro–Wilk test and visual inspection of Q–Q plots. Because qPCR bacterial load data were right-skewed and included zero values, bacterial counts were log10-transformed after adding 1 CFU before inferential analysis.

Differences in continuous site-level outcomes, including PPD and bacterial load, were analyzed using linear mixed-effects models with site type as a fixed effect. In every model, a random intercept for the patient was specified to account for the non-independence of multiple sites sampled within the same individual, while site type (HI, PI, or TT) was entered as the fixed effect of interest; models were fitted by restricted maximum likelihood. Binary outcomes, including BoP, SuP, and taxon-detection prevalence, were analyzed using generalized linear mixed models with a binomial distribution and a logit link, using the same patient-level random intercept structure. Planned pairwise comparisons were performed between HI and TT and between PI and TT. Additional subgroup analyses were conducted according to smoking status and periodontal disease status. Because they were based on small strata—most notably only seven smokers—these subgroup analyses were regarded as exploratory and hypothesis-generating and were not adjusted for multiple comparisons.

For patient-level baseline comparisons, independent-samples *t*-tests were used for normally distributed continuous variables, Mann–Whitney U tests for non-normally distributed continuous variables, and chi-square or Fisher’s exact tests for categorical variables, as appropriate. All tests were two-sided, and *p* < 0.05 was considered statistically significant. To account for multiple testing across bacterial taxa, *p*-values were adjusted using the Benjamini–Hochberg false discovery rate procedure. Bonferroni correction was applied for post hoc pairwise comparisons where relevant.

## 3. Results

### 3.1. Clinical and Demographic Profile of the Study Population

Forty-three patients with a mean age of 54.5 years were included in the study, 51% were males and 49% were females. Out of the total enrolled patients, 19 individuals were diagnosed with periodontal disease, and 7 reported a history of smoking. A total of 100 dental implants were sampled, of which 74 were classified as healthy and 26 exhibited signs of peri-implantitis. The average functional loading duration for the implants was 3.9 years. Thirty-seven implants had been in function for less than 5 years, while 63 had been functionally loaded for more than 5 years. According to the anatomical distribution, 44 implants were located in the maxilla and 56 in the mandible. Out of the 50 natural teeth sampled, 19 were diagnosed with periodontal disease, while 31 demonstrated a healthy periodontal status. Molars (n = 66), premolars (n = 60), and incisors (n = 24) were evenly represented across healthy implant (HI), peri-implantitis (PI), and natural tooth (TT) samples, ensuring a clinically relevant distribution, as described in Table 1.

### 3.2. Probing Depth Analysis by Site and Inflammatory Markers (BOP, SUP)

Probing depth analysis by site and inflammatory markers (BOP, SUP) revealed that the lingual site had the highest mean probing pocket depth (3.75 mm), followed by the distal (2.98 mm), buccal (2.93 mm), and mesial (2.85 mm) sites in descending order. PI sites exhibited greater probing depths across all anatomical locations compared with HI, with the lingual surfaces presenting the highest mean probing depths (8.00 mm), underscoring their vulnerability and emphasizing the importance of incorporating site-specific assessment and targeted hygiene protocols in peri-implant maintenance strategies.

The majority of PPD measurements (1–5 mm) were recorded in the healthy implant and natural tooth groups. In contrast, elevated PPD values between 5 and 8 mm were predominantly associated with peri-implantitis (PI). Importantly, no HI sites exhibited PPD values exceeding 5 mm. TT cases largely remained within the clinically acceptable PPD range. The lingual (L) site exhibited the highest BoP prevalence, at 75%. Buccal and distal sites showed moderate BoP rates of 38.57% and 32.73%, respectively. In contrast, mesial sites recorded the lowest BoP rate, at 30%. Lingual surfaces exhibited the highest prevalence of suppuration (50%), followed by buccal and mesial surfaces, each at around 20%. Distal sites showed slightly lower suppuration rates, at approximately 16%.

#### BoP and SuP by Sampled Element Type

This analysis demonstrates substantial variation in bleeding-on-probing (BoP) rates across clinical categories. PI sites exhibited a high BoP prevalence of 96.15%, underscoring its strong association with active inflammation and progressive tissue degradation. In contrast, HI sites showed BoP in 28.38% of cases, suggesting early or subclinical inflammatory activity. TT samples displayed the lowest BoP rate, at 18.00%, reflecting comparatively healthier periodontal conditions. Suppuration was predominantly associated with PI sites, occurring in 96.15% of cases. The element-level distribution of BoP and SuP is summarized in Table 2.

### 3.3. Quantitative Assessment of Periodontal Pathogens in Periimplantitis and Adjacent Teeth

#### 3.3.1. Comparison of Bacterial Load Between PI and TT Sites

In the primary comparison between peri-implantitis (PI) and adjacent tooth (TT) sites, linear mixed-effects models were fitted to log10-transformed bacterial counts, accounting for clustering within patients. A total of 26 PI sites and 50 TT sites were included. Compared with TT, PI sites showed significantly higher bacterial loads, as summarized in Table 3 No taxon showed a significantly higher bacterial load at TT sites after False Discovery Rate correction.

#### 3.3.2. Comparison of Bacterial Load Between HI and TT Sites

For the comparison between healthy implant (HI) and TT sites, 74 HI sites and 50 TT sites were analyzed using the same modeling approach. No taxon remained statistically significant after False Discovery Rate correction. The strongest unadjusted association was observed for *Prevotella intermedia* (Pi), which showed higher load at HI than at TT sites (β = 0.651, 95% CI 0.176 to 1.125, raw *p* = 0.0072), but this did not remain significant after correction for multiple testing (False Discovery Rate -adjusted *p* = 0.159).

#### 3.3.3. Comparison of Mean Bacterial Loads Between PI Sites and TT in Non-Smokers

**Figure 1:**
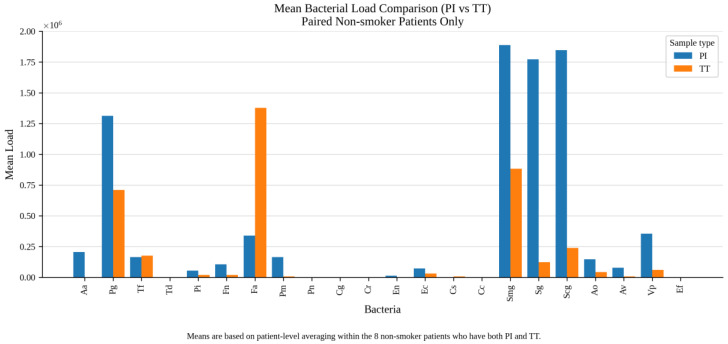
Mean bacterial load in PI vs. TT among non-smokers.

A comparison of mean bacterial loads between PI sites and TT samples from teeth in non-smokers may indicate site-specific differences and is presented in Figure 1. Several taxa showed a clear PI predominance, including *Aggregatibacter actinomycetemcomitans*, *Porphyromonas gingivalis*, *Fusobacterium nucleatum*, *Parvimonas micra*, *Eubacterium nodatum*, *Eikenella corrodens*, *Actinomyces odontolyticus*, *Actinomyces viscosus*, and *Veillonella parvula*, with the *Streptococcus constellatus group* and *Streptococcus gordonii* were also associated with PI. By contrast, *Filifactor alocis* was present in higher loads in TT than in PI. *Tannerella forsythia* exhibited no differences between the two groups, and other taxa are present in very low quantities. Nevertheless, the small number of smokers (n = 7) precluded any meaningful comparisons.

#### 3.3.4. Comparison of Mean Bacterial Loads Between PI Sites and TT Samples in Patients with Periodontal Disease

**Figure 2:**
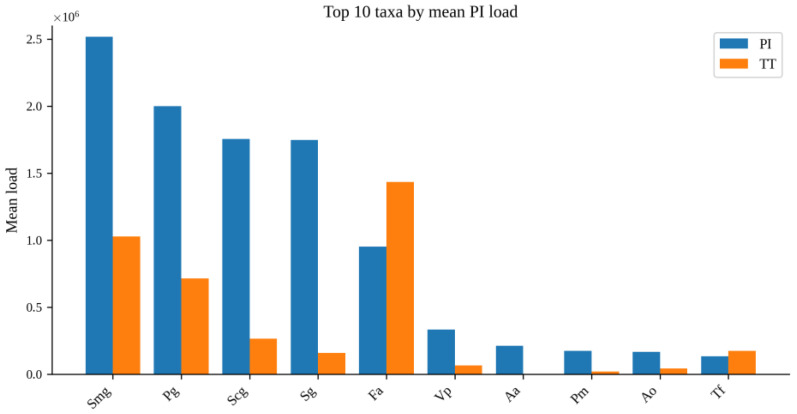
Mean bacterial load in PI vs. TT among patients with periodontal disease.

The taxa with the highest mean loads in PI were the *Streptococcus mitis group*, *Porphyromonas gingivalis*, *the Streptococcus constellatus group*, and *Streptococcus gordonii*, which together constituted the dominant microbial components, as shown in Figure 2. These taxa, together with *Aggregatibacter actinomycetemcomitans*, were present at substantially higher levels in PI than in TT. Additional taxa, including *Veillonella parvula*, *Aggregatibacter actinomycetemcomitans*, *Parvimonas micra*, and *Actinomyces odontolyticus*, also demonstrated consistently higher loads in PI, although at lower absolute levels. In contrast, *Filifactor alocis* exhibited higher mean abundance in TT, representing the most notable exception, while *Tannerella forsythia* showed a modest increase in TT. Overall, the data indicate that PI had a higher total bacterial burden and dominance of a limited number of high-abundance taxa, whereas TT display reduced levels for most organisms.

#### 3.3.5. Comparison of Mean Bacterial Loads Between PI Sites and TT Samples in Patients Without Periodontal Disease

**Figure 3:**
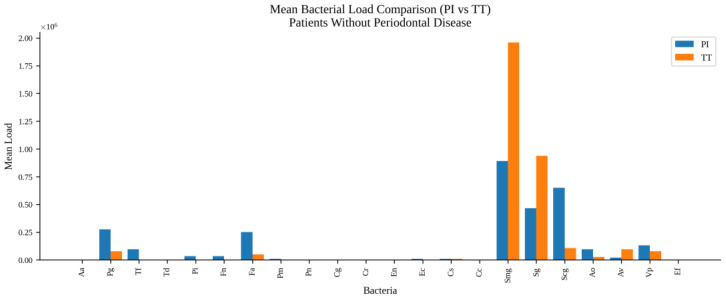
Mean bacterial load in PI vs. TT among patients without periodontal disease.

In patients without periodontal disease, the highest mean bacterial loads were observed for the *Streptococcus mitis group*, *Streptococcus gordonii*, and the *Streptococcus constellatus group*. As shown in Figure 3, among these, the *Streptococcus mitis group* showed the greatest overall abundance and was substantially higher in TT than in PI, whereas the *Streptococcus constellatus group* was higher in PI. Beyond these dominant taxa, PI showed higher mean loads for several organisms, including *Aggregatibacter actinomycetemcomitans*, *Porphyromonas gingivalis*, *Tannerella forsythia*, *Filifactor alocis*, *Actinomyces odontolyticus*, and *Veillonella parvula*. In contrast, TT exceeded PI mainly for the *Streptococcus mitis group*, *Streptococcus gordonii*, and *Actinomyces viscosus*. Most of the remaining taxa were present at very low mean levels in both groups, with only small intergroup differences.

### 3.4. Quantitative Assessment of Periodontal Pathogens in HI and TT

#### 3.4.1. Comparison of Mean Bacterial Loads Between HI Sites and TT Samples Among Non-Smokers

**Figure 4:**
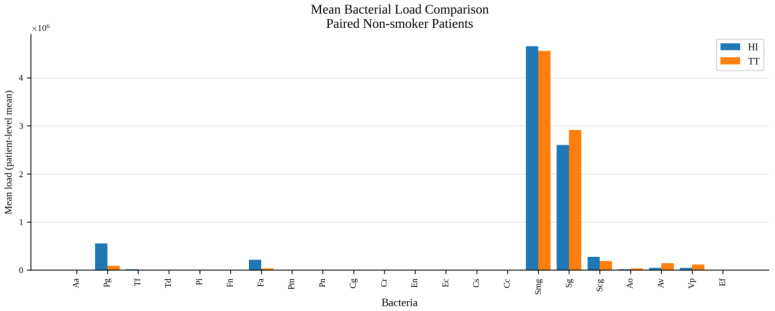
Mean bacterial load in HI vs. TT among non-smokers.

Figure 4 shows that the overall bacterial profile was broadly comparable between HI and TT sites, although several taxa showed site-related quantitative differences. The highest absolute bacterial loads in both groups were observed for Streptococcus mitis and *Streptococcus gordonii*. The *Streptococcus mitis group* appeared similar between HI and TT, whereas *Streptococcus gordonii* tended to be higher in TT. In contrast, *Porphyromonas gingivalis* and *Filifactor alocis* showed visibly higher mean loads in HI than in TT, with smaller HI-predominant differences also evident for several additional taxa, including the *Streptococcus constellatus group*. A limited number of organisms, such as *Actinomyces viscosus* and *Veillonella parvula*, appeared relatively more abundant in TT. Taken together, the graphic suggests that HI and TT share a common ecological background but differ in the relative burden of selected bacterial species.

#### 3.4.2. Comparison of Mean Bacterial Loads Between HI Sites and TT Samples in Patients Without Periodontal Disease

**Figure 5:**
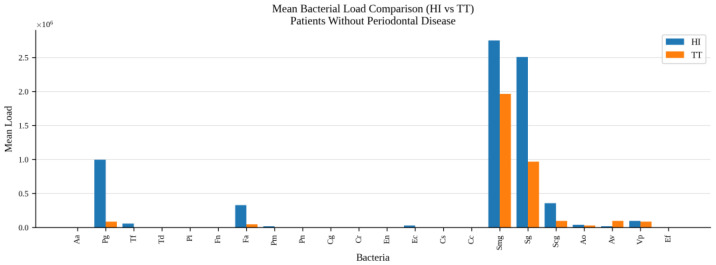
Mean bacterial load in HI vs. TT among patients without periodontal disease.

The analysis in Figure 5. showed that mean bacterial loads were generally higher at healthy implant sites (HI) than at adjacent tooth sites (TT). The greatest differences were seen for the *Streptococcus mitis group*, *Streptococcus gordonii*, and *Porphyromonas gingivalis*, with additional elevations at HI for *Filifactor alocis* and the *Streptococcus constellatus group*. Most other taxa were present at very low mean levels in both groups. *Actinomyces viscosus* was one of the few taxa showing a slightly higher mean load at TT than at HI. Other organisms were detected at low levels in both groups, indicating limited microbial complexity in the absence of disease.

#### 3.4.3. Comparison of Mean Bacterial Loads Between HI Sites and TT Samples in Patients with Periodontal Disease

**Figure 6:**
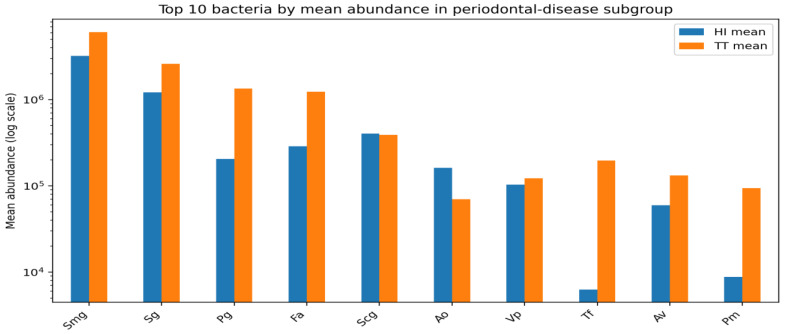
Mean bacterial load in HI vs. TT among patients with periodontal disease.

In Figure 6, on the other hand, in the periodontal-disease subgroup, TT showed higher mean abundance than HI for most taxa, including the *Streptococcus mitis group*, *Streptococcus gordonii*, *Porphyromonas gingivalis*, *Filifactor alocis*, and *Tannerella forsythia*. In contrast, HI enrichment was limited, with higher levels observed mainly for *Actinomyces odontolyticus*, while the *Streptococcus constellatus group* remained comparable between groups. Overall, TT was associated with a greater bacterial burden, whereas HI exhibited a more constrained microbial profile.

### 3.5. Patient-Level Prevalence of Detected Subgingival Bacterial Taxa

#### 3.5.1. Heatmaps According to Periodontal Status

When comparing the prevalence of each taxon at the individual-patient level between PI and TT sites using a patient-clustered logistic model, no taxon remained statistically significant after FDR correction. The strongest unadjusted association was observed for the *Streptococcus constellatus group*, with higher odds of detection at PI sites than at TT sites (OR = 4.597, 95% CI: 1.642 to 12.874, raw *p* = 0.0037), although this did not remain significant after FDR adjustment (adjusted *p* = 0.081).

**Figure 7:**
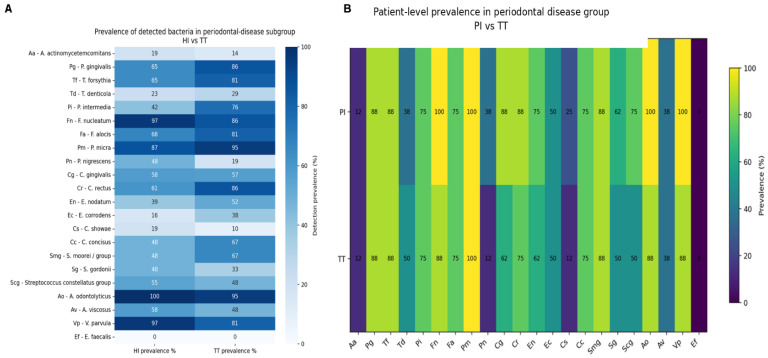
Prevalence of detected subgingival bacteria across clinical site types within the periodontal-disease subgroup (HI vs. TT—panel (**A**); PI vs. TT—panel (**B**)).

No taxon showed a statistically significant difference in prevalence between HI and TT after FDR correction. The lowest raw *p*-value was observed for *Tannerella forsythia* (OR = 1.996, 95% CI: 1.007 to 3.956, raw *p* = 0.0477), but this association was not significant after multiple-testing correction (FDR-adjusted *p* = 0.613). These associations are illustrated in Figure 7 where cell color reflects detection prevalence and annotated values indicate prevalence percentages where shown.

In Panel A, TT sites demonstrated high prevalence for multiple classical periodontal taxa, including *Porphyromonas gingivalis*, *Tannerella forsythia*, *Treponema denticola*, *Prevotella intermedia*, *Filifactor alocis*, *Parvimonas micra*, *Campylobacter rectus*, *Eubacterium nodatum*, *Eikenella corrodens*, *Campylobacter concisus*, and the *Streptococcus mitis group*, supporting the concept that adjacent teeth may serve as an important microbial reservoir. In contrast, HI sites showed higher prevalence for *Aggregatibacter actinomycetemcomitans*, *Fusobacterium nucleatum*, *Prevotella nigrescens*, *Campylobacter gracilis*, *Capnocytophaga species*, *Streptococcus gordonii*, the *Streptococcus constellatus group*, *Actinomyces odontolyticus*, *Actinomyces viscosus*, and *Veillonella parvula*. In Panel B, TT sites maintained a prevalence profile broadly comparable to that observed at PI sites, supporting the view that adjacent teeth remain microbiologically relevant when periodontal disease is present.

#### 3.5.2. Heatmaps According to the Non-Smoking Subgroup

**Figure 8:**
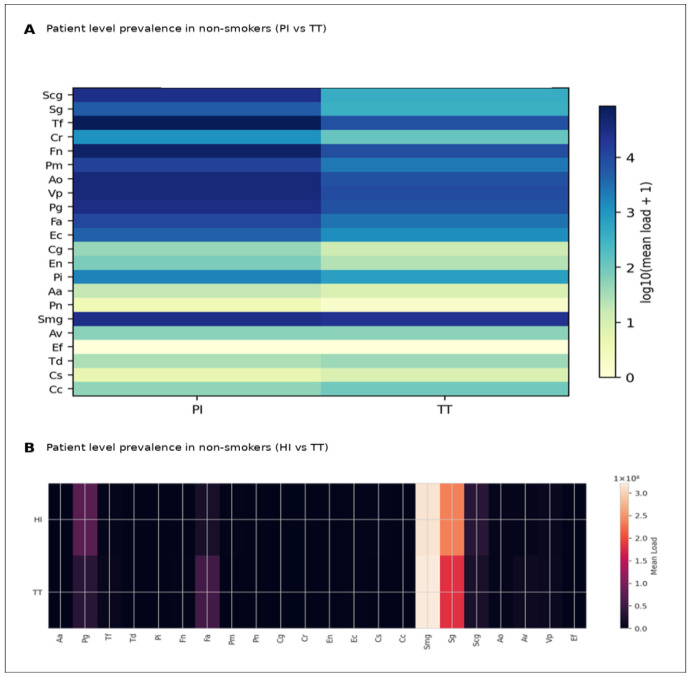
Prevalence of detected subgingival bacteria across clinical site types within the non-smokers subgroup (PI vs. TT—panel (**A**); HI vs. TT—panel (**B**)).

Figure 8 displays the detection-prevalence heatmaps for the non-smoking subgroup. PI generally exhibited higher detection prevalence than TT across most taxaacross most taxa. HI and TT were dominated by a limited number of taxa, particularly *the Streptococcus mitis group*, *Streptococcus gordonii*, and the *Streptococcus constellatus group*, which showed the highest detection prevalence in both site types. *Porphyromonas gingivalis* appeared more prominent at HI and *Filifactor alocis* at TT sites.

Ten taxa—*Campylobacter rectus*, *Porphyromonas gingivalis*, the *Streptococcus constellatus group*, *Fusobacterium nucleatum*, *Eubacterium nodatum*, *Filifactor alocis*, *Parvimonas micra*, *Tannerella forsythia*, *Actinomyces odontolyticus*, *Treponema denticola*—showed significantly elevated bacterial loads at PI sites compared to TT sites, all with positive β values and FDR-adjusted *p* < 0.05; yet not a single taxon reached significance in the prevalence comparisons. These bacteria were commonly found at both PI and TT sites but existed in higher quantities at PI sites and mapped almost exactly onto established periodontal pathogen complexes:

- Red complex: *Porphyromonas gingivalis* (Pg), *Tannerella forsythia* (Tf), *Treponema denticola* (Td).
- Orange complex/associated: *Fusobacterium nucleatum* (Fn), *Parvimonas micra* (Pm), *Eubacterium nodatum* (En), *Campylobacter rectus* (Cr), *Filifactor alocis* (Fa), *the Streptococcus constellatus group* (Scg), and *Actinomyces odontolyticus* (Ao).

*The Streptococcus constellatus group* shows the largest effect size (β = 2.080), suggesting that it may be a particularly relevant marker of the PI microbiome.

HI are microbiologically indistinguishable from TT. Every single HI vs. TT comparison is non-significant—both for load and prevalence. The β values for HI are consistently small and scattered around zero (ranging from −0.831 to 0.675).

Taxa at the bottom of the table (*Campylobacter gracilis*, *Prevotella nigrescens*, *Veillonella parvula*, *Actinomyces viscosus*, *Campylobacter concisus*, *Streptococcus gordonii*, the *Streptococcus mitis group*, *Capnocytophaga species*, *Aggregatibacter actinomycetemcomitans*, *Eikenella corrodens*, and *Enterococcus faecalis*) show no significant differences in any comparison and generally small effect sizes, consistent with them being commensal or peripheral members of the subgingival community. The combined bacterial-load and detection-prevalence findings are summarized in Table 4.

### 3.6. Mean Socransky Complex Load by Oral Site, Smoking Status, and Periodontal Disease

**Figure 9:**
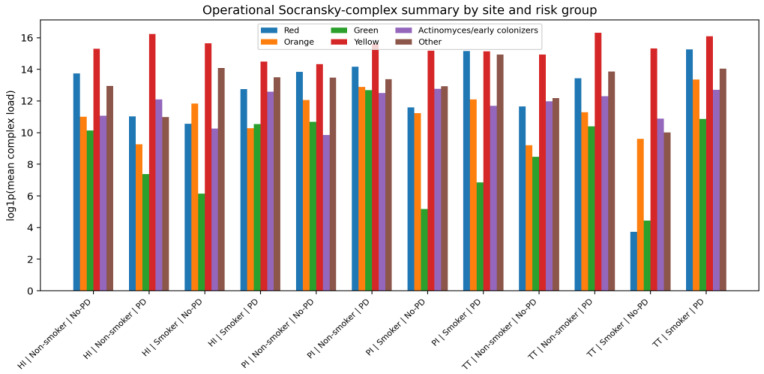
Operational Socransky-complex bacterial load by clinical site type and patient risk group.

The yellow complex displayed the highest complex load and remained consistently elevated across smoking and periodontal categories. The green complex showed the lowest load overall and the greatest between-group variation. Visual inspection suggested higher complex loads in the PD than the No-PD group at several sites, particularly at PI and TT, whereas differences at HI appeared less consistent. Smoking-related patterns were also heterogeneous across sites, with lower green complex loads observed in several smoker/No-PD groups, while smokers with PD often showed similar or higher loads for the red and orange compared with non-smokers with PD. A notable exception was the TT smoker/No-PD group, which showed a markedly reduced red complex load relative to the other groups. The corresponding complex-level pattern is shown in Figure 9.

## 4. Discussion

Peri-implantitis is one of the most common biological complications associated with dental implants, with a reported prevalence ranging from 19 to 25% at the patient level and 12–18% at the implant level [11]. As such, there is considerable interest in understanding its microbial etiology.

Current evidence describes peri-implantitis as a multifactorial dysbiotic disease that arises from the combined influence of the subgingival biofilm, the host inflammatory response, and implant- and patient-related risk factors, rather than from a simple quantitative increase in any single group of organisms [12]. The quantitative differences reported here should therefore be interpreted within this broader ecological and host-modulated framework.

Recent peri-implantitis literature [7,13,14] has consistently shown substantial microbiological similarity between diseased implant sites and adjacent natural teeth, suggesting a shared core microbiota and possible microbial exchange [15,16]. In agreement with this concept, the present study showed broad overlap between peri-implant and tooth sites; however, the clearest distinction was quantitative rather than qualitative. Compared with adjacent teeth, PI sites showed significantly higher bacterial loads for 10 taxa, but no prevalence comparison remained significant after multiple-testing correction. Taken together, these findings indicate that, in this cohort, peri-implantitis was characterized primarily by an increased burden of disease-associated bacteria rather than by the consistent emergence of unique taxa.

The load-based enrichment observed at PI sites is consistent with previous microbiological studies. Classical periopathogens such as *Porphyromonas gingivalis*, *Tannerella forsythia*, *Treponema denticola*, and *Fusobacterium nucleatum* have repeatedly been associated with peri-implantitis [17,18,19] and in the present study, these pathogens showed significantly higher loads at PI sites. The additional enrichment of *Filifactor alocis* and *Parvimonas micra* is sustained by recent molecular evidence [20,21] indicating that peri-implant dysbiosis includes other anaerobic taxa beyond the traditional red-complex organisms. Therefore, our results support the view that peri-implantitis shares major microbiological features with periodontitis while differing mainly in the magnitude of pathogen accumulation at the diseased implant site, a pattern that is consistent with, but cannot by itself establish microbial sharing between periodontal and peri-implant sites.

The microbial pattern at peri-implantitis sites is consistent with a meta-analysis showing associations between peri-implantitis and *P. gingivalis*, *T. forsythia*, *T. denticola*, *F. nucleatum*, *and P. intermedia* [7]. The most abundant taxa in our samples belonged to the red (*Porphyromonas gingivalis*, *Tannerella forsythia*, *Treponema denticola*) and orange complexes described by Socransky et al. [22]. This suggests that, in our cohort, peri-implantitis is not driven by colonization of implants by a new microbial community [13,14].

The higher levels of *Filifactor alocis* and *Parvimonas micra* at PI sites are consistent with the growing evidence that these bacteria are involved in PI. *F. alocis* is a Gram-positive anaerobe that has several features that may help it contribute to disease, including the ability to tolerate oxidative stress, interfere with the host’s innate immune response, and increase the virulence of *P. gingivalis* in polymicrobial biofilms [21]. Pyrosequencing studies have also shown that *Fusobacterium*, *Parvimonas*, and other related anaerobic genera are more abundant in peri-implant biofilms [20].

In contrast, the overall comparison between HI and TT was less distinct. No taxon showed significant differences in bacterial load or prevalence after multiple-testing correction, indicating that healthy implants and adjacent teeth shared largely comparable microbial profiles. These findings align with observations of the predominance of Gram-positive facultative species, particularly Streptococcus and Actinomyces, in peri-implant sites, with substantial overlap with the microbiota of natural teeth [18].

Although peri-implant and tooth sites belong to the same oral ecosystem, they do not represent biologically identical niches. Molecular studies conducted under different periodontal conditions support the view that a shared oral ecosystem can nevertheless yield site-specific microbial configurations [13,23]. In contrast to natural teeth, implants lack cementum and the periodontal ligament, and the peri-implant surface topography created to improve osseointegration may facilitate bacterial adhesion and retention [13,24]. They may also have a limited blood supply, with reduced migration of immune cells into the peri-implant sulcus. In addition, the supracrestal collagen fibers are distributed around the implant rather than attaching perpendicularly (as they do around teeth), thereby weakening the barrier against bacterial invasion [24]. These anatomical and ecological differences may help explain why, once inflammation is established, peri-implant sites can support higher pathogen loads even when the detected taxa are broadly similar to those found at adjacent teeth.

The findings related to the adjacent teeth are clinically relevant. In subjects with periodontal disease, TT prevalence patterns were closer to PI than to HI, and even in the absence of periodontal disease, the microbial profiles of PI and TT still showed clear overlap. This agrees with previous evidence reported in a meta-analysis of 12 prospective cohort studies, showing that a history of periodontitis is a major risk factor for peri-implantitis and marginal bone loss [8] and supporting the hypothesis that adjacent teeth may act as microbial reservoirs, a relationship that, in the present cross-sectional setting, is best interpreted as a microbiological association between periodontal and peri-implant sites rather than as evidence of transfer in either.

However, because the present study was cross-sectional and both sites were sampled at the same time point, it cannot determine the direction of transmission. Previous studies have shown that bacteria may be shared between natural teeth and implants. Botero et al. [25] found a microbiological overlap between peri-implant mucosal lesions and adjacent teeth in partially edentulous patients. Aoki et al. [26] showed that the presence of *A. actinomycetemcomitans*, *P. intermedia*, *P. gingivalis*, *T. denticola*, and *F. nucleatum* in the peri-implant sulcus was linked to their presence in adjacent teeth rather than in contralateral teeth. Zhuang et al. [27] also reported that periodontal pathogens were found at both periodontal and peri-implant sites in the same subjects, regardless of clinical status. Our findings of a broad overlap between PI and adjacent TT sites are consistent with these results and support the idea that implants and neighboring teeth are not separate microbiological compartments but rather ecologically connected niches that share a substantial portion of their microbiota. Because sampling was simultaneous and cross-sectional, these observations should be read as associations; they do not establish that organisms move between the two niches or in which direction any such exchange might occur.

The subgroup analyses suggest that smoking and periodontal status may modulate microbial patterns, but these findings should be interpreted with caution due to the small number of patients. Nevertheless, the broader increase in *Porphyromonas gingivalis*, *Filifactor alocis*, *Treponema denticola*, *Tannerella forsythia*, *Campylobacter rectus*, *Actinomyces odontolyticus*, and *Eubacterium nodatum* at PI sites in smokers is in line with the deep-sequencing study by Tsigarida et al., which showed that smoking can shift the peri-implant microbiome toward a more pathogen-rich and less diverse community, even around clinically healthy implants [28], with smoking acting as a local factor that influences biofilm composition.

In non-smokers, some of the largest differences involved *Streptococcus constellatus group* and other taxa, with *Filifactor alocis* occasionally appearing at higher levels in TT. Likewise, in patients without periodontal disease, TT showed higher levels of health-associated streptococci. These patterns suggest that host and local conditions may shape the expression of dysbiosis, but the dominant signal remained consistent across analyses: PI was associated with higher pathogenic load, not with an entirely separate microbiota. The *Streptococcus constellatus* group showed the biggest difference between PI and TT sites. Although it is usually considered part of the normal oral flora, *S. constellatus* has often been detected in subgingival sites in chronic and refractory periodontitis, and is also known for its ability to form abscesses [29]. Its role in peri-implant infection has received limited attention so far. Given the clear difference observed in our study, the S. constellatus group may be a more important contributor to peri-implant dysbiosis than currently recognized, and should be examined more closely in future studies as a potential disease-related marker.

Socransky complex analysis is consistent with this interpretation. The persistently high yellow-complex load across categories and the greater variability of the green, orange, and red complexes suggest that some components of the oral microbiota remain relatively stable, whereas disease-related changes are driven by selective expansion of pathogenic consortia. Overall, the present findings support a model in which peri-implantitis develops within a shared oral microbial ecosystem but is distinguished by a stronger inflammatory response and a more pronounced quantitative dysbiotic shift at the implant site. The increased bacterial load documented here should not be read as implying that peri-implantitis reduces to a simple increase in microbial numbers. Recent comprehensive reviews emphasize that peri-implantitis is a multifactorial dysbiotic condition in which microbial colonization interacts with host susceptibility and immune response, biomechanical overload, implant surface and prosthetic characteristics, and systemic and lifestyle factors such as smoking and diabetes [12,24]. The quantitative shifts observed in this study are therefore better understood as one measurable component of a broader dysbiotic and host-mediated process: the diseased site is characterized not only by an increase in certain organisms but also by an altered ecological balance within a susceptible host. This framing is consistent with our own subgroup observations, in which smoking and periodontal status appeared to modulate the microbial pattern. Related pilot data from patients with aggressive periodontitis also indicate that peri-implant and periodontal microbial diversity may vary with the underlying periodontal context [30].

This study has several limitations. First, its cross-sectional design precludes conclusions regarding temporality, disease progression, or causality. Second, the microbiological assessment was restricted to a targeted qPCR panel of 22 taxa, which does not capture the full breadth of the subgingival microbiome. Although qPCR allowed precise quantification of 22 pre-specified periodontal taxa, this targeted approach cannot capture non-targeted, fastidious, or as-yet-uncultured organisms. Recent sequencing- and metagenomics-based studies indicate that the peri-implant microbiome is substantially more diverse, and broader molecular methods would be required to characterize it fully.

Third, sampling was performed at one site per implant or tooth, selected as the deepest site, thereby standardizing site-level comparisons but not representing a full-mouth or full-surface assessment. Selecting the deepest site was intended to maximize the likelihood of detecting dysbiotic change, but this strategy may overrepresent inflamed conditions and introduce a potential site-selection bias that limits generalizability to the dentition as a whole. Fourth, the subgroup analyses stratified by smoking and periodontal status rested on small strata—most notably only seven smokers—and are best regarded as exploratory. Finally, the implant-to-tooth ratio reflects the sampling protocol, which prioritized implant sites and included adjacent teeth as the within-subject comparator, rather than the cohort’s dentition status; the sampled teeth should not be taken to represent a full periodontal assessment of each patient.

Within these limitations, the present findings support a substantial microbiological overlap between peri-implant and adjacent tooth sites while indicating that peri-implantitis is characterized primarily by increased bacterial burden rather than by consistent prevalence-based differences. Longitudinal studies using broader microbiome approaches are needed to clarify whether these quantitative shifts precede peri-implant disease or result from the inflammatory process.

## 5. Conclusions

Peri-implantitis and adjacent teeth exhibited substantial microbiological overlap, but peri-implantitis was distinguished primarily by higher loads of selected disease-associated taxa and markedly greater clinical inflammation. Healthy implant and tooth sites were broadly similar overall. The data suggest that peri-implantitis represents a site-specific quantitative overgrowth of a well-defined set of classical periodontal pathogens, rather than the acquisition of a novel microbial community.

## Figures and Tables

**Table 1:** Demographic and clinical characteristics of the study population.

Characteristic	Value
Patients, n	43
Age, mean (years)	54.5
Sex, male/female (%)	51/49
Patients with periodontal disease, n	19
Smokers, n	7
Implants sampled, n	100
Healthy implants (HI), n	74
Peri-implantitis (PI), n	26
Mean functional loading (years)	3.9
Functional loading, <5/≥5 years (n)	37/63
Implant location, maxilla/mandible (n)	44/56
Natural teeth sampled, n	50
Periodontally diseased/healthy teeth (n)	19/31
Site position, molar/premolar/incisor (n)	66/60/24

**Table 2:** BoP and SuP by sampled element type.

Analysis Type	Total Cases	Positive BoP	BoP Rate (%)	Positive Suppuration	Suppuration Rate (%)
HI	74	21	28.38	2	2.70
PI	26	25	96.15	25	96.15
TT	50	9	18.00	4	8.00

**Table 3:** Bacterial load in PI.

Bacterium	β	95% CI	Raw *p*	FDR-Adjusted *p*
*Campylobacter rectus* (Cr)	1.292	0.773–1.811	1.08 × 10^−6^	2.38 × 10^−5^
*Porphyromonas gingivalis* (Pg)	1.374	0.730–2.019	2.93 × 10^−5^	3.22 × 10^−4^
*Streptococcus constellatus group* (Scg)	2.080	1.013–3.148	1.34 × 10^−4^	9.79 × 10^−4^
*Fusobacterium nucleatum* (Fn)	1.125	0.471–1.779	7.48 × 10^−4^	0.004
*Eubacterium nodatum* (En)	1.581	0.604–2.557	0.0015	0.006
*Filifactor alocis* (Fa)	1.405	0.531–2.279	0.0016	0.006
*Parvimonas micra* (Pm)	1.186	0.394–1.979	0.0033	0.010
*Tannerella forsythia* (Tf)	1.553	0.466–2.640	0.0051	0.012
*Actinomyces odontolyticus* (Ao)	0.795	0.241–1.349	0.0049	0.012
*Treponema denticola* (Td)	1.397	0.356–2.437	0.0085	0.019

**Table 4:** Summary of bacterial load and prevalence comparisons between implant and tooth sites.

Taxon	PI vs. TT Load β (adj *p*)	HI vs. TT Load β (adj *p*)	PI vs. TT Prevalence OR (adj *p*)	HI vs. TT Prevalence OR (adj *p*)
Cr	1.292 (2.36 × 10^−5^)	0.175 (0.787)	5.066 (0.452)	0.914 (0.924)
Pg	1.374 (0.000322)	0.07 (0.895)	1.705 (0.452)	0.842 (0.768)
Scg	2.08 (0.000979)	0.518 (0.489)	4.597 (0.081)	1.424 (0.613)
Fn	1.125 (0.004)	0.105 (0.822)	3.691 (0.466)	0.883 (0.924)
En	1.581 (0.006)	0.373 (0.489)	1.67 (0.466)	1.538 (0.613)
Fa	1.405 (0.006)	0.675 (0.489)	3.129 (0.452)	1.491 (0.613)
Pm	1.186 (0.01)	0.131 (0.787)	2.613 (0.581)	0.829 (0.912)
Tf	1.553 (0.012)	0.538 (0.479)	1.98 (0.466)	1.996 (0.613)
Ao	0.795 (0.012)	−0.022 (0.969)	—	0.597 (0.797)
Td	1.397 (0.019)	0.445 (0.489)	2.063 (0.452)	1.635 (0.613)
Cg	0.435 (0.161)	−0.064 (0.895)	1.378 (0.777)	1.46 (0.75)
Pn	0.309 (0.161)	0.143 (0.489)	1.183 (0.784)	1.083 (0.924)
Vp	0.744 (0.161)	0.237 (0.722)	2.561 (0.557)	1.487 (0.778)
Av	−0.818 (0.257)	−0.533 (0.489)	0.521 (0.515)	0.863 (0.912)
Pi	0.537 (0.257)	0.651 (0.159)	0.674 (0.466)	1.453 (0.613)
Cc	0.352 (0.333)	−0.445 (0.422)	0.861 (0.794)	0.507 (0.613)
Sg	0.631 (0.414)	0.388 (0.722)	1.296 (0.751)	1.127 (0.912)
Smg	−0.633 (0.414)	−0.831 (0.479)	0.585 (0.466)	0.656 (0.613)
Cs	−0.311 (0.414)	−0.153 (0.787)	0.69 (0.466)	0.715 (0.687)
Aa	0.148 (0.445)	0.109 (0.612)	0.87 (0.777)	1.456 (0.678)
Ec	−0.017 (1)	−0.265 (0.722)	0.808 (0.784)	0.707 (0.747)
Ef	0 (1)	0 (1)	—	—

Note. β > 0 indicates a higher bacterial load at the implant group than at TT. OR > 1 indicates higher odds of detection in the implant group than in TT. Values are presented as effect estimates with FDR-adjusted *p*-values in parentheses—indicates not estimable.

## Data Availability

The original contributions presented in this study are included in the article. Further inquiries can be directed to the corresponding author.
